# Genomic divergence and phenotypic heterogeneity of *Legionella longbeachae* reveal a putative novel proinflammatory serogroup with genome reduction and mobilome expansion

**DOI:** 10.1128/aem.01109-26

**Published:** 2026-09-22

**Authors:** Jun-Wei Xu, Yuan-Tong He, Xuefu Zhou, Xiao-Yong Zhan

**Affiliations:** 1 Scientific Research Center, The Seventh Affiliated Hospital, Sun Yat-sen University 582261, Shenzhen, China; 2 Center for Digestive Medicine, The Seventh Affiliated Hospital, Sun Yat-sen University 582261, Shenzhen, China; Royal Botanic Gardens Kew, Surrey, United Kingdom

**Keywords:** *L. longbeachae*, pan-genome, genome reduction, serogroup 3, host-pathogen interaction, virulence

## Abstract

*Legionella longbeachae* is an emerging respiratory pathogen primarily found in soil environments, yet its genomic architecture and evolutionary strategies remain poorly characterized. Here, through pan-genomic and functional analysis of 242 *L. longbeachae* isolates, we reveal an open pan-genome driven by extensive horizontal gene transfer and marked functional divergence between core and accessory genomes. We identify a phylogenetically distinct lineage, designated as putative serogroup 3 (sg3), recovered from Chinese environments. This lineage diverges from canonical serogroups 1 and 2 through genome reduction (mean size 4.01 Mb), absence of plasmids, and a truncated O-antigen biosynthesis cluster lacking a key N-acetyltransferase-encoding gene (orf9). *In vitro* infection models across multiple human cell lines show that sg3 elicits increased host cell death and elevated proinflammatory cytokine transcription in epithelial cells compared to sg1 and sg2. Phylogenetically corrected association analyses revealed no significant link between this phenotype and individual accessory virulence factors. Instead, rank-transformed phylogenetically generalized least squares (PGLS) regression demonstrated a significant positive association with expansion of the mobilome (COG X), while intracellular trafficking (COG U) and defense (COG V) repertoires were contracted. These findings reveal a distinct pathogenic profile in sg3 that differs from the canonical serogroups, characterized by heightened inflammatory activation rather than immune evasion. Our work advances the understanding of *L. longbeachae* population structure, challenges the reductionist utility of traditional serogrouping, and highlights the potential need for revised diagnostic considerations as well as continued surveillance of lineages displaying enhanced cytotoxicity and proinflammatory responses, which may be associated with mobilome expansion.

**IMPORTANCE:**

*Legionella longbeachae* is an understudied yet emerging cause of Legionnaires’ disease, with a distinct soil-based ecology. Using comparative pan-genomics and functional infection assays across 242 isolates (including 39 newly sequenced from China), we identify a putative novel serogroup (sg3) that has undergone marked genome reduction (4.01 Mb) and completely lost plasmids, contrasting with near-ubiquitous plasmid carriage in sg1/sg2. Despite its streamlined genome, putative sg3 exhibits enhanced cytotoxicity in all tested human cell lines and elevated proinflammatory cytokine expression, particularly in epithelial cells. These phenotypes are not explained by individual virulence genes but are significantly associated with mobilome (COG X) expansion. Our findings question the assumption that genome reduction necessarily diminishes pathogenic potential and suggest that higher-order genomic restructuring, potentially linked to mobilome expansion, may be associated with increased inflammatory responses. Furthermore, they highlight the potential need for continued surveillance and consideration of revised diagnostic approaches to include this rare but possibly underdiagnosed lineage.

## INTRODUCTION

Legionnaires’ disease (LD), a severe and potentially fatal form of atypical pneumonia, remains a persistent global public health challenge (1). While *Legionella pneumophila* (*L. pneumophila*) causes the majority of documented outbreaks, *Legionella longbeachae* (*L. longbeachae*) has emerged as a clinically and ecologically distinct pathogen, particularly endemic in Australia and New Zealand, with increasing reports across Southeast Asia and Europe (2–4). Unlike the aquatic niche adaptation of *L. pneumophila*, *L. longbeachae* predominantly thrives in soil environments (5–7), especially compost, potting soils, and garden mulches, creating unique exposure pathways for horticultural workers, landscapers, and immunocompromised individuals. Clinically, *L. longbeachae* infections present with classic LD manifestations, including high fever, pulmonary consolidation, neurological involvement, and gastrointestinal distress. However, accumulating clinical and immunological evidence suggests that *L. longbeachae* may elicit distinct host immune responses (8, 9), exhibit differential tissue tropism, and display variable disease severity compared to *L. pneumophila*, yet the molecular and genomic underpinnings of these differences remain poorly defined.

The taxonomic classification and epidemiological tracking of *L. longbeachae* have historically relied on molecular serogrouping, primarily based on sequence variation in the macrophage infectivity potentiator (*mip*) gene (10). Currently, the species is divided into two recognized serogroups (sg1 and sg2), with sg1 accounting for the overwhelming majority of human infections globally (11). Conventional serotyping, however, may not fully capture the extensive genomic plasticity and functional heterogeneity inherent to environmental *Legionella* populations. Recent comparative genomic studies have established that *Legionella* species possess highly open pan-genomes shaped by recurrent horizontal gene transfer, mobile genetic element proliferation, and homologous recombination (12, 13). Yet, whether the recognized serogroup framework captures the true extent of *L. longbeachae* diversity remains uncertain, particularly as emerging environmental isolates increasingly fall outside established sg1/sg2 boundaries. The drivers of lineage-specific divergence in host cell invasion, intracellular replication, and immunomodulatory capacity remain unclear. This divergence could arise from the acquisition or loss of discrete virulence effectors or alternatively from higher-order genomic architectures, including mobilome expansion, O-antigen cluster remodeling, and adaptive genome reduction.

Resolving this question requires integrated comparative genomics, functional annotation, and host-pathogen assays within a phylogenetically rigorous framework. While comprehensive catalogs of *Legionella* virulence determinants have been curated for *L. pneumophila* (14), the distribution, evolutionary dynamics, and phenotypic impact of these factors across *L. longbeachae* lineages are poorly characterized. Moreover, conventional genotype-phenotype association approaches frequently confound true functional relationships with phylogenetic non-independence, yielding spurious correlations that misattribute lineage history to adaptive trait evolution (15). Phylogenetically corrected models are, therefore, essential to disentangle vertical inheritance from convergent or lineage-specific adaptation. Furthermore, functional stratification of genomic content may offer a more predictive framework for understanding pathoadaptive strategies than binary presence/absence patterns of individual accessory genes. This is particularly true for the enrichment of mobilome-associated (COG X), intracellular trafficking (COG U), and defense (COG V) gene categories.

In this study, we present pan-genomic and functional investigation of 242 *L. longbeachae* genomes, integrating 39 newly sequenced environmental isolates with 203 high-quality public assemblies. We delineate the population structure, O-antigen cluster diversity, and functional composition of the core and accessory genomes, identifying a distinct lineage recovered from Chinese environments that exhibits pronounced genome reduction coupled with mobilome enrichment. Using integrative O-antigen cluster architecture analysis, *mip* allelic profiling, and relatedness analysis, we resolve this lineage as genetically distinct from classical sg1 and sg2 isolates, designating it as a putative novel serogroup (sg3). Through systematic *in vitro* infection assays across multiple human cell types, we demonstrate marked phenotypic heterogeneity in internalization efficiency, host cell cytotoxicity, and proinflammatory cytokine induction. By integrating phylogenetically generalized least squares (PGLS) regression with functional profiling, we find that phenotypic divergence is not strongly linked to the differential distribution of individual accessory virulence genes but is instead significantly associated with coordinated genomic restructuring and mobilome expansion. Our findings broaden the current understanding of *L. longbeachae* pathoadaptation and provide genomic and functional correlations that point toward potential mechanistic hypotheses for the heightened inflammatory phenotype of this lineage.

## RESULTS

### Pan-genome analysis of *L. longbeachae* reveals an open, functionally heterogeneous species

**Fig 1:**
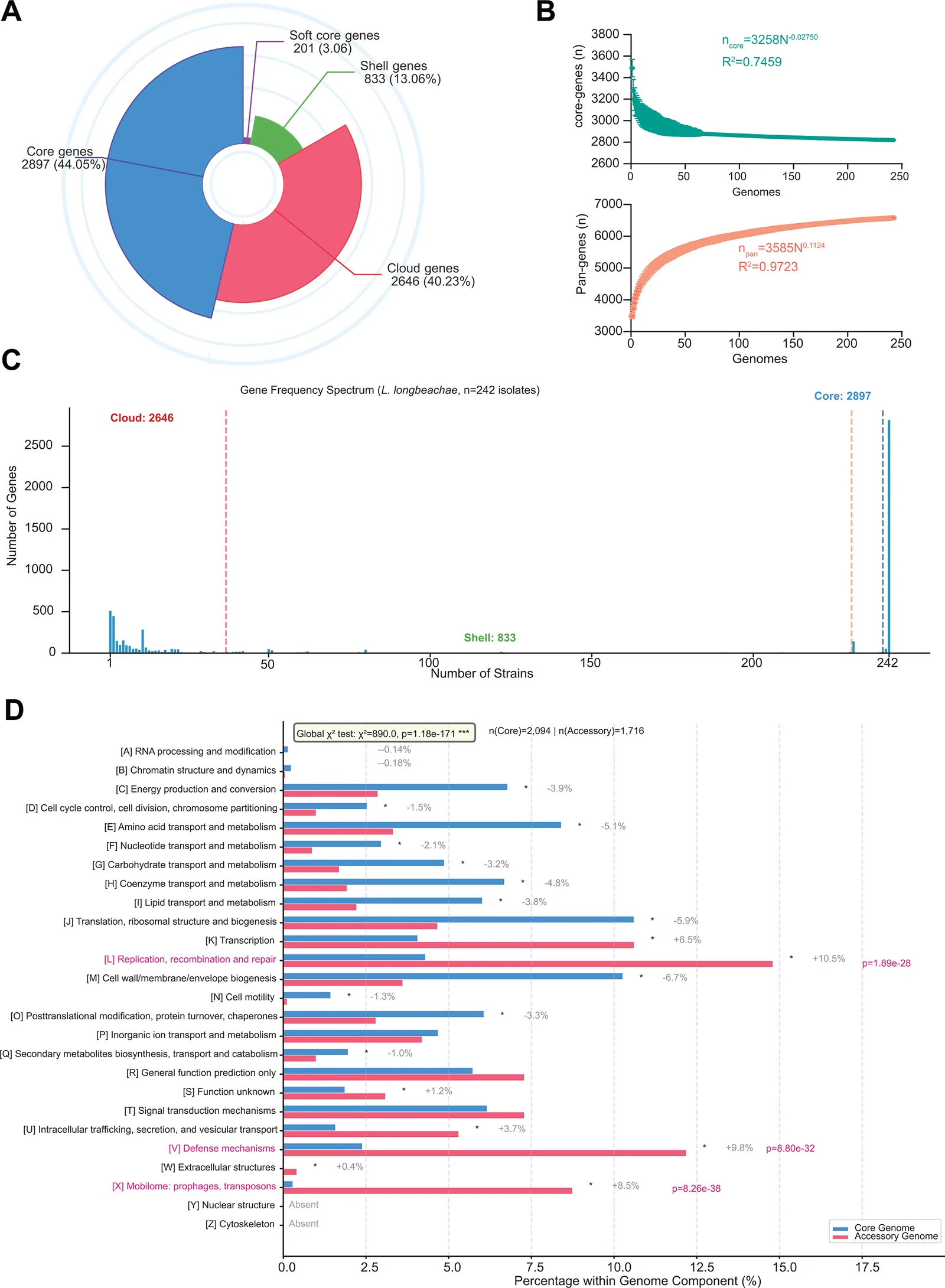
Pan-genome structure and functional architecture of *L. longbeachae*. (**A**) Composition of the pan-genome, partitioned into core, soft-core, shell, and cloud gene sets. Cloud genes account for > 40% of the total pan-genome. (**B**) Pan-genome (orange) and core-genome (cyan) accumulation curves as a function of the number of input genomes. The continuously expanding pan-genome (*r* = 0.11) contrasts with the rapidly saturating core genome. (**C**) Gene frequency distribution histogram across the pangenome, showing a heavily skewed distribution dominated by low-frequency accessory genes. (**D**) Functional classification of core and accessory genes based on COG categories. The accessory genome is enriched in genes involved in replication/recombination/repair (L), mobilome (X), and defense mechanisms (V).

The pan-genome was partitioned into core, soft-core, shell, and cloud gene sets, with cloud genes present in fewer than 15% of isolates, accounting for over 40% of the total genetic repertoire, indicative of pronounced genomic polymorphism (Fig. 1A). Analysis of pan-genome dynamics showed that the core genome rapidly saturated, whereas the pan-genome exhibited continuous expansion with the sequential addition of genomes. The calculated openness parameter was γ = 0.11, consistent with an open pan-genome model (Fig. 1B). This was independently corroborated by the gene frequency distribution, which displayed a skewed, long-tail distribution dominated by rare, low-copy genes (Fig. 1C), further confirming a high degree of genomic fluidity. To investigate the functional basis of this diversity, we compared Clusters of Orthologous Groups (COG) functional profiles between core and accessory gene sets of *L. longbeachae*. The core genome was predominantly populated by housekeeping functions such as metabolism and translation, whereas the accessory genome was significantly enriched in genes associated with replication, recombination, and repair (category L); mobilome: prophages and transposons (category X); and defense mechanisms (category V) (Fig. 1D). Collectively, these findings characterize *L. longbeachae* as a highly heterogeneous species possessing an open pan-genome with extensive functional compositional diversity.

### Genomic heterogeneity reveals a distinct lineage within *L. longbeachae*

**Fig 2:**
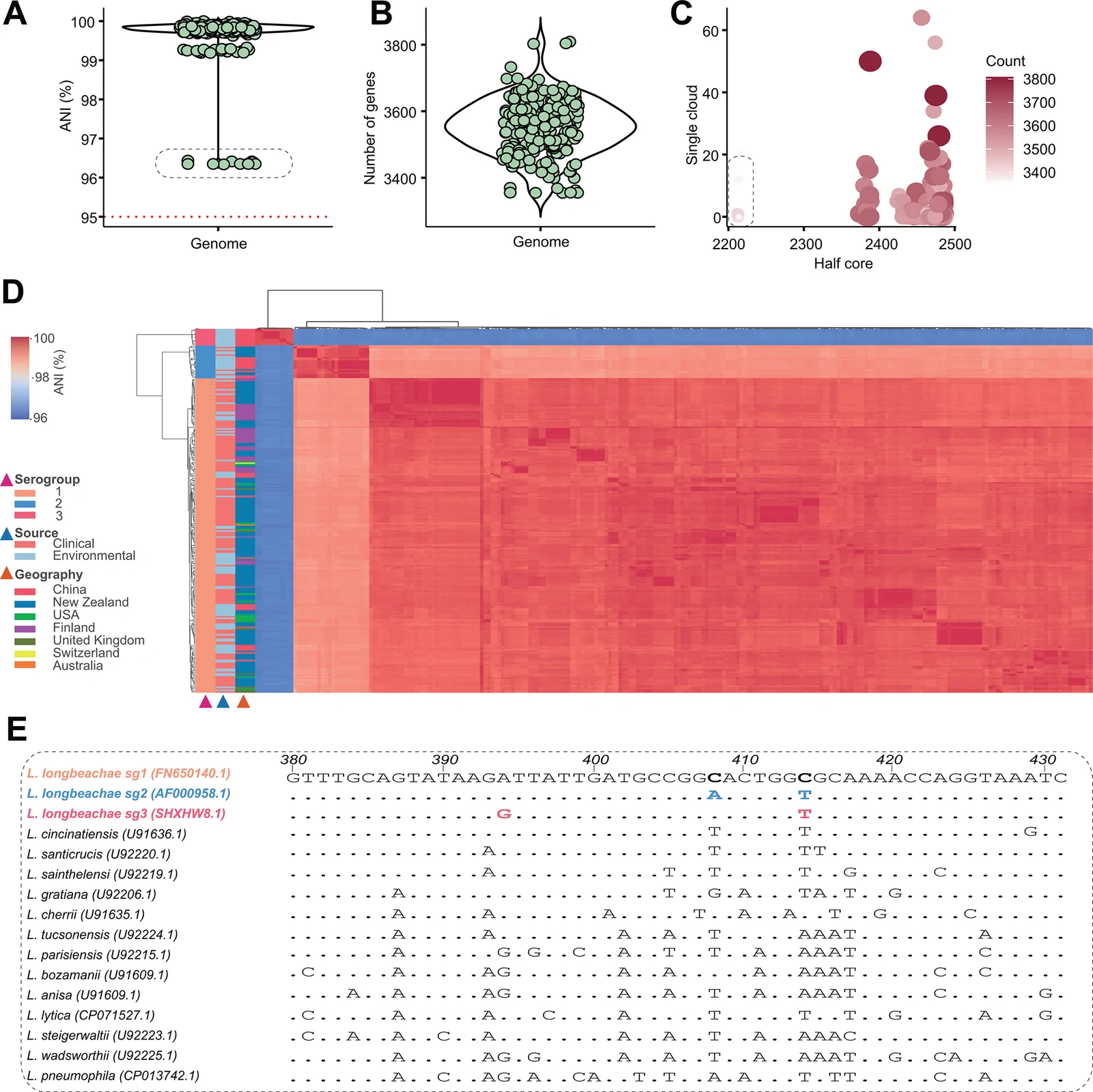
Genomic population structure and phylogenetic delineation of *L. longbeachae* isolates. (**A**) ANI-based clustering of 242 *L. longbeachae* genomes relative to the reference strain NCTC11477 (GenBank accession: GCA_004283175.1). Three discrete genetic groups are resolved, with a distinct cluster of 11 Chinese environmental isolates (dashed box). (**B**) Distribution of coding sequence counts across the collection. (**C**) Segregation of isolates based on conserved (half-core) gene content. (**D**) Genome-wide genetic relatedness (ANI) of 242 isolates; 231 isolates with known serogroup status partition into sg1 and sg2, whereas the 11 Chinese environmental isolates form a distinct, unrelated cluster. (**E**) *mip* gene polymorphism analysis. The 380–431 bp fragment containing *L. longbeachae* serogroup-discriminatory SNPs was aligned for representative strains. Species names and accession numbers are indicated on the left; dots denote identity to the sg1 reference. Eleven Chinese isolates harbor *mip* polymorphisms distinct from sg1/sg2, indicating a unique molecular serotype.

Initial quality assessment and comparative genomics against the reference strain NCTC11477 revealed significant genomic plasticity. Average nucleotide identity (ANI) distributions revealed a structured population comprising three genetically distinct clusters (Fig. 2A). Genome sizes varied considerably, encoding between 3,300 and 3,800 predicted proteins, and the ratio of “half-core” genes (present in ~50% of isolates) versus “single-cloud” genes (unique to single isolates) mirrored this tripartite subdivision (Fig. 2B and C).

Detailed pairwise ANI analysis of all 242 isolates confirmed that the 231 isolates (203 from public database and 28 from China) with assigned serogroup status partitioned strictly into sg1 and sg2, whereas the 11 Chinese environmental isolates formed a discrete, phylogenetically divergent cluster (Fig. 2D), suggesting a distinct lineage. To investigate the basis of this stratification, we examined the *mip* gene, an established molecular marker for *L. longbeachae* serogroup differentiation (10). Eleven environmental isolates recovered from Shanghai and Chuzhou, China, harbored *mip* polymorphisms that were incongruent with either sg1 or sg2 (Fig. 2E).

### O-antigen cluster divergence supports designation of a putative novel serogroup

**Fig 3:**
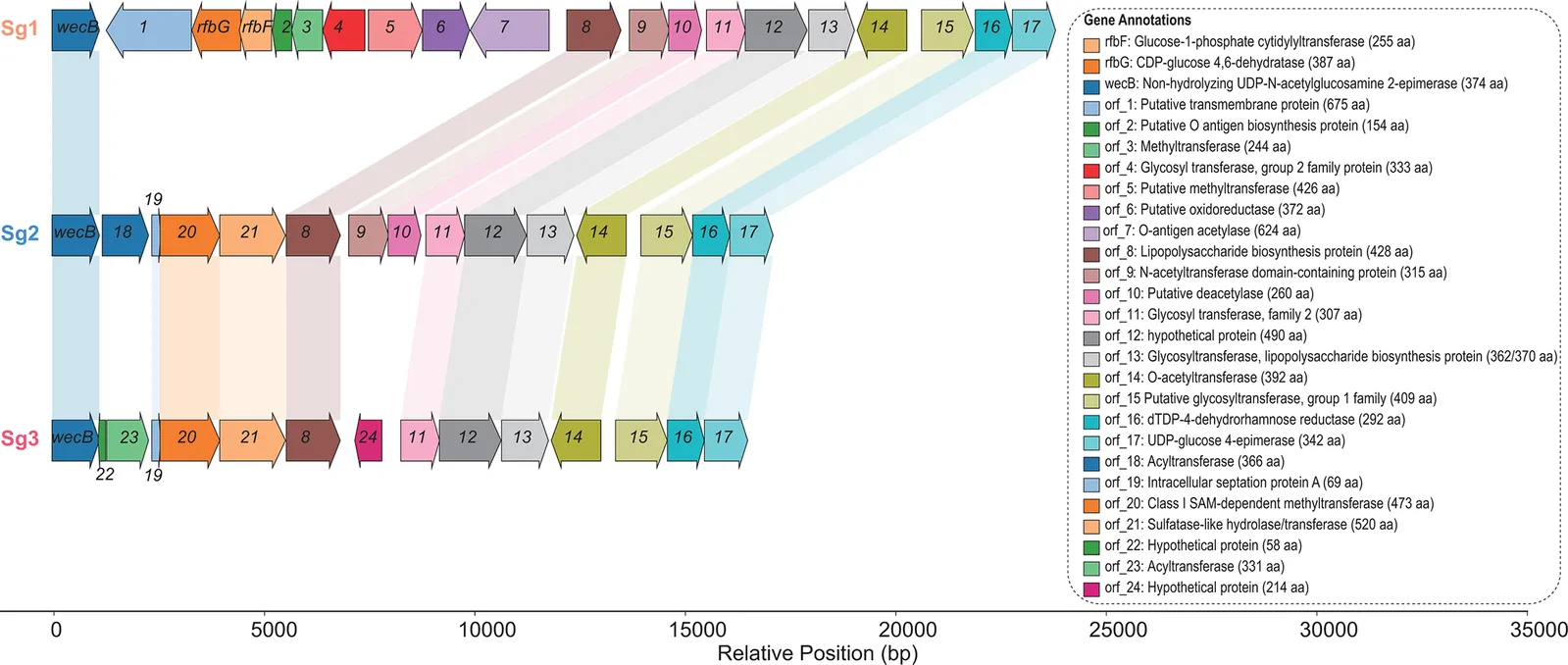
Organization and comparison of the O-antigen biosynthetic gene clusters. The sg1 locus spans 23.8 kb (20 ORFs), the sg2 locus spans 17.1 kb (15 ORFs), and the novel cluster spans 16.5 kb (15 ORFs). The novel cluster uniquely lacks orf9, which encodes a putative N-acetyltransferase. The isolates in the novel cluster are tentatively designated serogroup 3 (sg3).

Serogroup specificity in *Legionella* is generally recognized to be principally defined by lipopolysaccharide O-antigen variation (16). Comparative analysis of the O-antigen biosynthetic locus identified 27 genes in *L. longbeachae*, including *rfbF*, *rfbG*, and *wecA*, as well as 24 additional open reading frames (ORFs) designated orf1-orf24. The sg1 locus spanned 23.8 kb and contained 20 ORFs, whereas the sg2 locus spanned 17.1 kb and harbored 15 ORFs. The novel cluster occupied 16.5 kb and likewise contained 15 ORFs; however, it uniquely lacked orf9, which encodes a putative N-acetyltransferase that may be involved in O-antigen biosynthesis (Fig. 3). The convergence of a distinct *mip* profile, genetic relatedness, and O-antigen locus architecture, specifically the absence of orf9, supports the designation of these 11 isolates as a putative novel serogroup, here termed putative serogroup 3 (sg3). Having confirmed that all 11 isolates share >96% ANI with the type strain NCTC11477 (Fig. 2A), which exceeds the 95%–96% species delineation threshold, we further validated their species assignment using *in silico* DNA–DNA hybridization (iDDH) as an independent measure. Pairwise iDDH values ranged from 70.6% to 70.9% (Table S2), all well above the 70% species boundary, unambiguously placing the 11 isolates within the *L. longbeachae* species. These results, together with the distinct O-antigen cluster architecture and *mip* allelic profile, support the designation of this lineage as a putative novel serogroup (sg3) rather than a separate species

### Phylogenomic distinctiveness and reductive genomic features of the putative sg3 lineage

**Fig 4:**
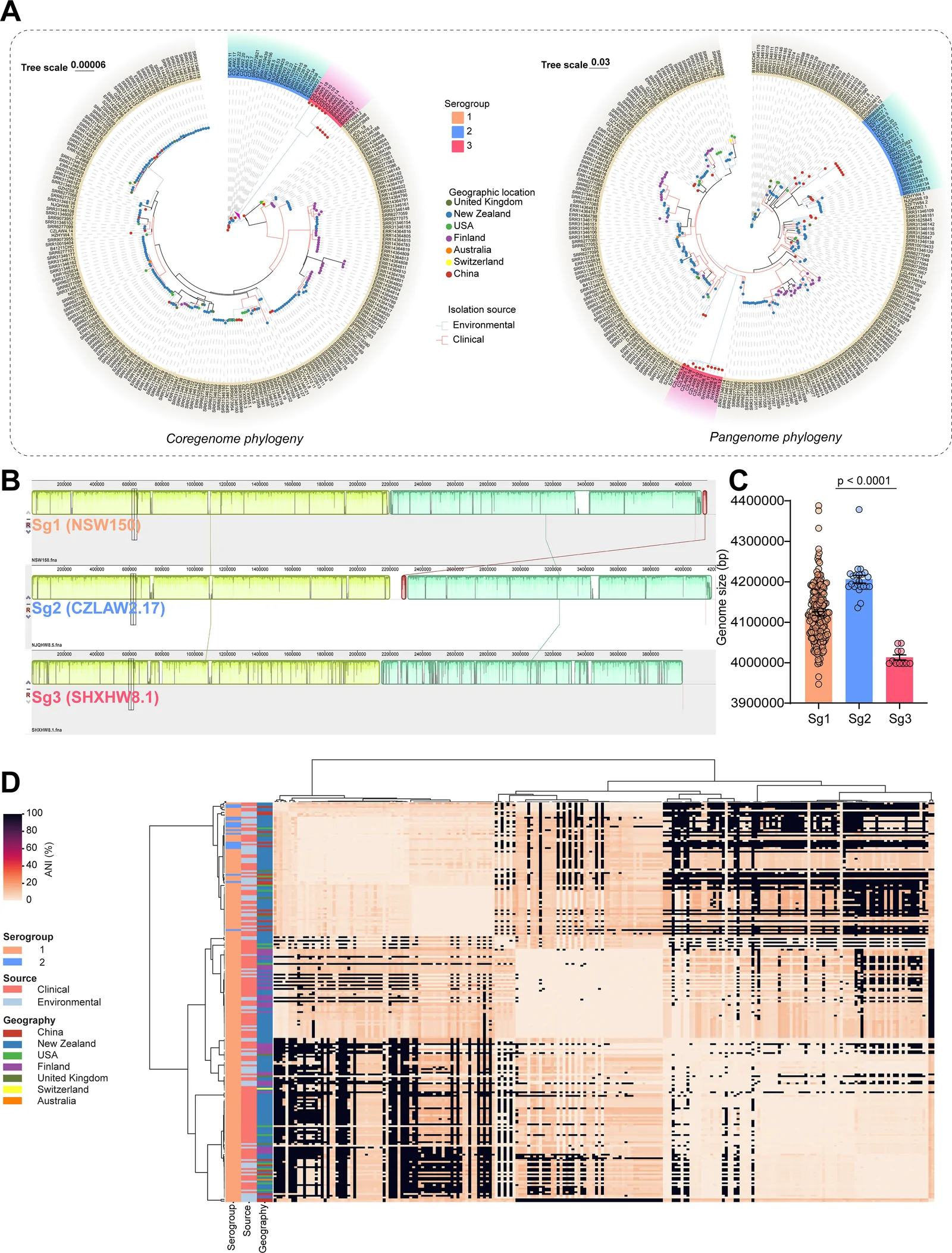
Phylogenomic positioning and genome reduction of the putative sg3 lineage.(**A**) Congruent core-genome (left) and pan-genome (right) phylogenies resolving *L. longbeachae* into three distinct clades. The sg3 branch (purple triangle) in the core-genome tree is compressed to 2.5% of its true length for visualization purposes. (**B**) Synteny comparison of representative isolates shows extensive collinearity but marked genome contraction in sg3. (**C**) Genome size distribution across the three sgs. Data are shown as bar plots overlaid with scatter dots, with each dot representing an individual isolate. The mean genome size of sg3 (4.01 Mb) is significantly smaller than that of sg1 (4.12 Mb) and sg2 (4.20 Mb). Data represent mean ± SEM. ****P < 0.0001 (one-way ANOVA). (**D**) Prevalence and ANI-based clustering of plasmids. Sg3 isolates lack plasmids; plasmids from sg2 cluster with those in a subset of sg1 isolates, without correlation to the geographic origin or isolation source (clinical vs environmental).

Having established the 11 environmental isolates as a candidate novel serogroup (sg3) through *mip* sequence variation and O-antigen locus architecture (Fig. 2 and 3 and), we next assessed their phylogenomic position and genomic architecture. Core-genome SNP-based and pan-genome-based phylogenies were congruent, resolving sg1, sg2, and sg3 as three discrete, well-supported clades (Fig. 4A). Sg3 formed a monophyletic cluster that was phylogenetically distant from sg1 and sg2, corroborating its discrete lineage status within *L. longbeachae*.

This phylogenetic divergence was coupled with marked reductive genome evolution. The reference strain NCTC11477 was excluded from synteny analysis because its genome assembly is not gap-free. Instead, we selected three complete-genome representatives: sg1 (NSW150), sg2 (CZLAW8.17), and putative sg3 (SHXHW8.1). Synteny analysis revealed extensive collinearity but clear contraction in the sg3 genome (Fig. 4B). Quantitatively, sg3 possessed a significantly smaller mean genome size (4.01 Mb) than sg1 (4.12 Mb) and sg2 (4.20 Mb) (one-way ANOVA, *P* < 0.0001; Fig. 4C). Screening for extrachromosomal elements showed that sg3 isolates lacked detectable plasmids, whereas the vast majority of sg1 isolates (208/209, 99.52%) and most sg2 isolates (16/22, 72.73%) harbored one or more plasmid sequences (Table S3). ANI-based clustering further revealed that sg2 plasmids grouped with those carried by a subset of sg1 isolates irrespective of the geographic origin or isolation source (Fig. 4D), suggesting active plasmid exchange between these two serogroups. In stark contrast, sg3 isolates carried no plasmids, suggesting that sg3 may be genetically isolated from sg1 and sg2 with respect to plasmid-mediated gene exchange. Collectively, these findings establish genome reduction and the absence of plasmids as defining genomic features of the putative sg3 lineage.

### Putative serogroup 3 exhibits enhanced cytotoxicity and proinflammatory capacity when compared with sg1 and sg2

**Fig 5:**
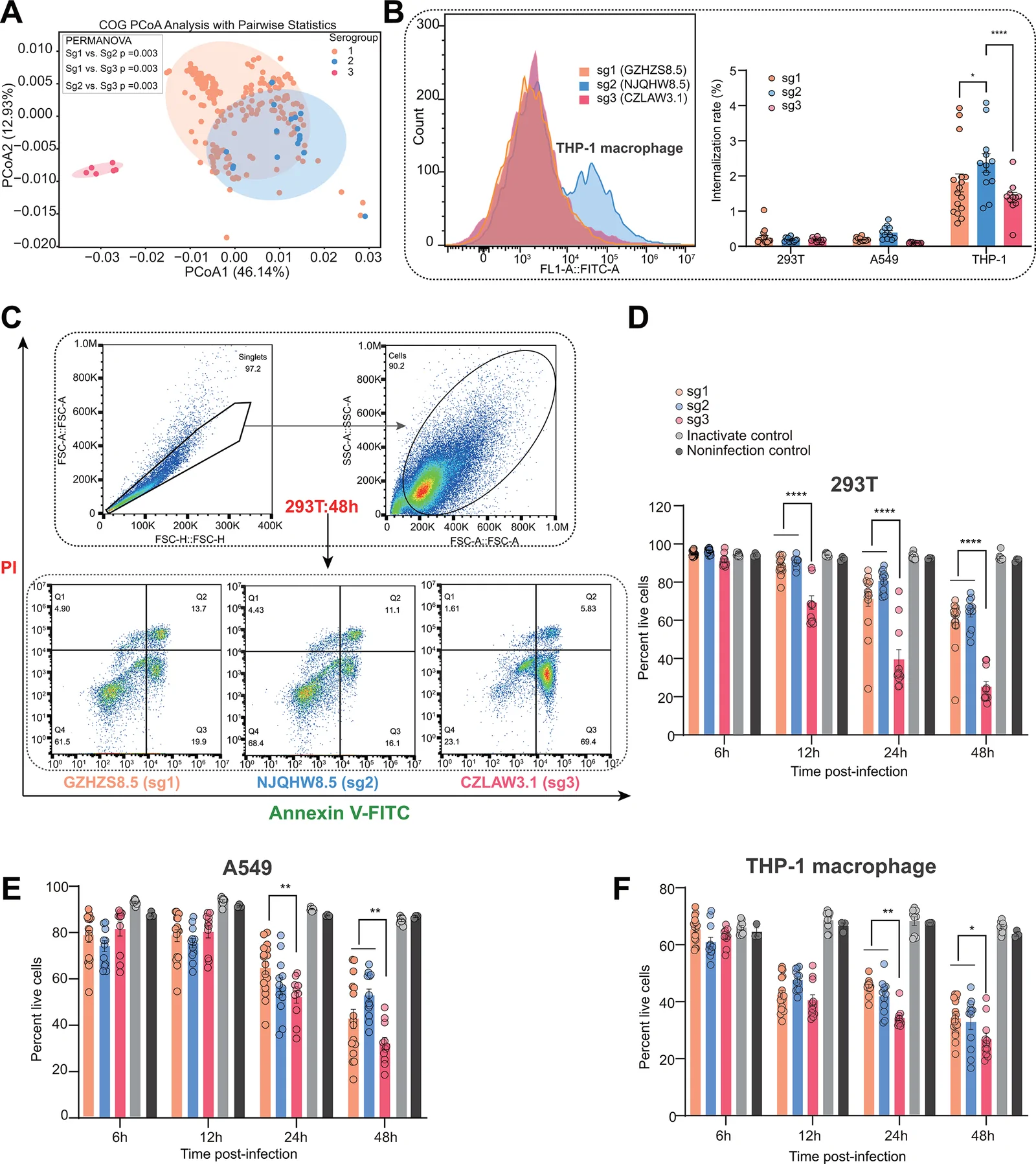
Functional divergence and enhanced cytotoxicity of the putative sg3 lineage. (**A**) PCoA based on COG functional category abundance profiles of 242 isolates. sg3 occupies a distinct functional space separated from sg1 and sg2 (PERMANOVA, *P* = 0.003 for all pairwise comparisons). (**B**) Internalization efficiency in THP-1 macrophages and in non-phagocytic (293T and A549) cells at 2 hours post-infection (hpi). (**C**) Representative flow cytometry plots of 293T cells at 48 hpi with three serogroup representative isolates: sg1 (GZHZS8.5), sg2 (NJQHW8.5), and sg3 (CZLAW3.1). Gating strategy: single cells were first selected to exclude doublets, followed by debris removal based on FSC-A/SSC-A. Live cell fractions were determined by annexin V/PI staining. (**D–F**) Quantification of host cell viability at 24 and 48 hpi in 293T (**D**), A549 (**E**), and THP-1 (**F**) cells infected with sg1, sg2, or sg3 isolates. sg3 infection consistently reduces viable cell fractions across all three cell lines compared to sg1 and sg2.

To determine whether the genomic streamlining of putative sg3 confers distinct host-interaction phenotypes, we first compared the functional gene profiles of all 242 isolates. Principal coordinate analysis (PCoA) based on COG category abundance revealed clear serogroup stratification, with sg3 occupying a functional space distinct from sg1 and sg2 (Fig. 5A). We, therefore, assessed serogroup-specific infection phenotypes across human macrophage-like (THP-1) and epithelial (293T and A549) cell lines. Internalization rates in epithelial cells were comparable across all three serogroups; however, in THP-1 cells, sg2 displayed significantly higher internalization efficiency than sg1 and sg3 (Fig. 5B).

Strikingly, sg3 infection induced pronounced host cell death across all three cell lines. At 24 and 48 h post-infection, sg3-exposed cultures showed a marked reduction in viable cells compared with sg1 and sg2 (Fig. 5C through F). This cytopathic effect was strictly dependent on live bacteria as heat-killed sg3 (70°C, 30 min, sterility was confirmed by plating on BCYE agar) failed to reduce cell viability, indicating that cell death resulted from active infection rather than from exposure to bacterial components. To exclude the possibility that serogroup-specific structural components (for example, lipopolysaccharide or peptidoglycan) drove the differential inflammatory responses, we measured cytokine transcription following exposure to heat-killed bacteria. Heat-killed preparations from all three serogroups induced only low-level cytokine responses (mean log₂ fold change <0.5 for most cytokines; Fig. S1), which were comparable across serogroups (Fig. S2). These results indicate that the enhanced inflammatory response to live sg3 cannot be recapitulated by heat-killed bacteria, suggesting a requirement for active bacterial processes (e.g., Dot/Icm secretion).

**Fig 6:**
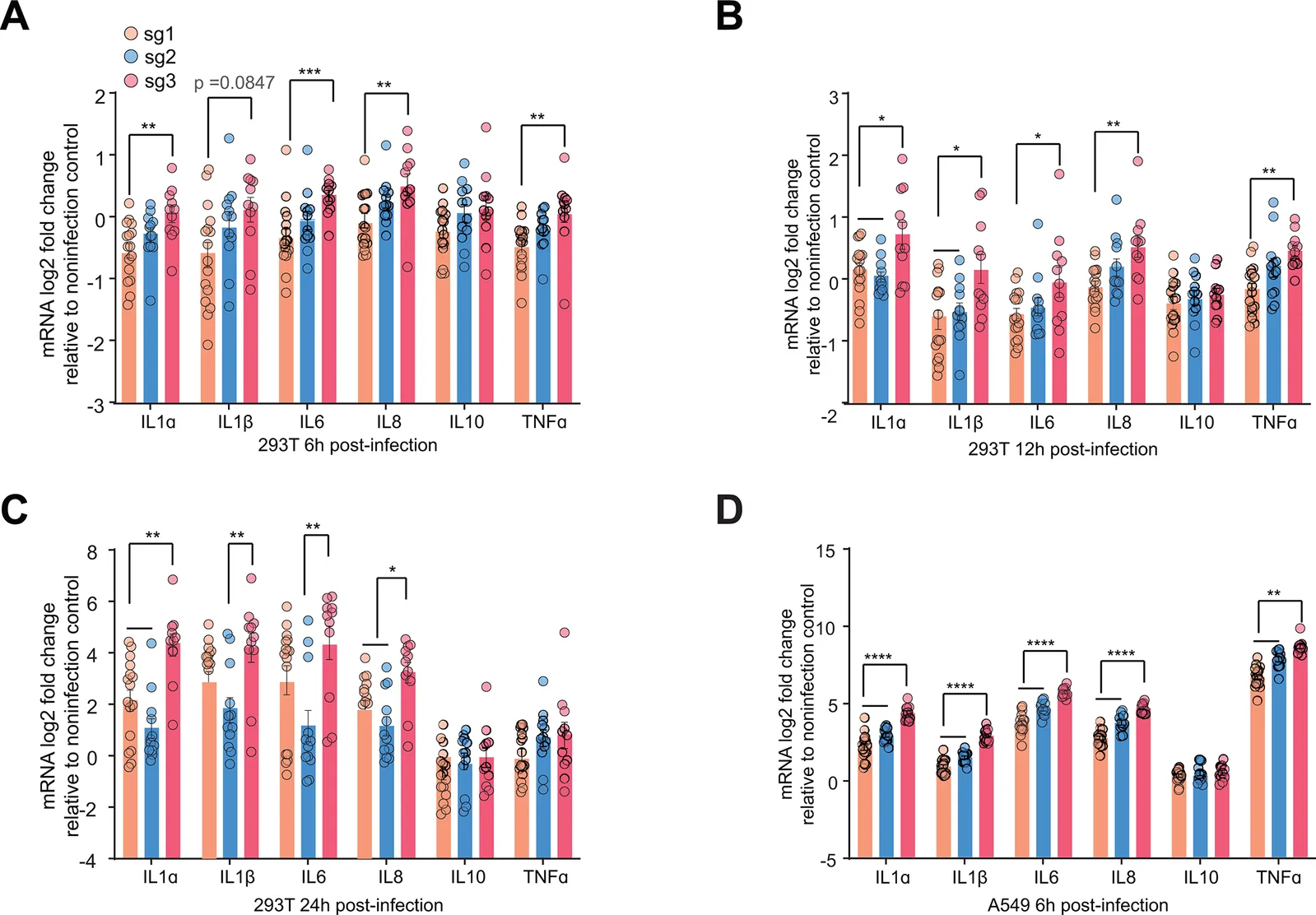
Serogroup-specific proinflammatory cytokine responses in epithelial cells. (**A–C**) qRT-PCR analysis of IL1α, IL1β, IL6, IL8, TNFα, and IL10 in 293T cells at 6, 12, and 24 hpi. (**D**) Cytokine expression in A549 cells at 6 hpi. Data are expressed as mean ± SEM of isolate-level means (sg1, *n* = 16; sg2, *n* = 12; putative sg3, *n* = 11). *P < 0.05; **P < 0.01; ***P < 0.001; ****P < 0.0001 (one-way ANOVA with Tukey’s *post hoc* test).

To further characterize the host response, we next assessed whether the enhanced cytotoxicity of sg3 is accompanied by a distinct inflammatory signature. In epithelial cells, live sg3 triggered a potent, serogroup-specific proinflammatory program. In 293T cells, sg3 consistently induced higher mRNA levels of IL1ɑ, IL1β, IL6, IL8, and TNFɑ across most time points when compared with sg1 or sg2. At 6 h and 12 h post-infection, all five cytokines were significantly upregulated when compared with sg1 or sg2 or both; at 24 h, significant elevation was maintained for IL1ɑ, IL1β, IL6, and IL8, whereas TNFɑ showed a consistent but nonsignificant increase. In contrast, IL10 remained at baseline throughout the time course (Fig. 6A through C). In A549 cells, significant cytokine induction by sg3 was restricted to the early stage (6 h) (Fig. 6D). In A549 and THP-1 cells, IL10 tended to be lower following live sg3 infection relative to heat-killed controls at select time points, although the functional significance of this observation remains unclear. These phenotypic differences were not explained by differential intracellular proliferation as bacterial growth rates were comparable among serogroups across all cell models (Fig. S3A and B). Moreover, THP-1 macrophages showed no significant serogroup-dependent cytokine divergence (Fig. S3C), and A549 cells exhibited no cytokine differences at later time points (12–48 h) (Fig. S3C), indicating that the enhanced cytotoxic and inflammatory responses are cell type- and serogroup-specific.

### Functional genomic correlates of serogroup-specific host-interaction phenotypes

**Fig 7:**
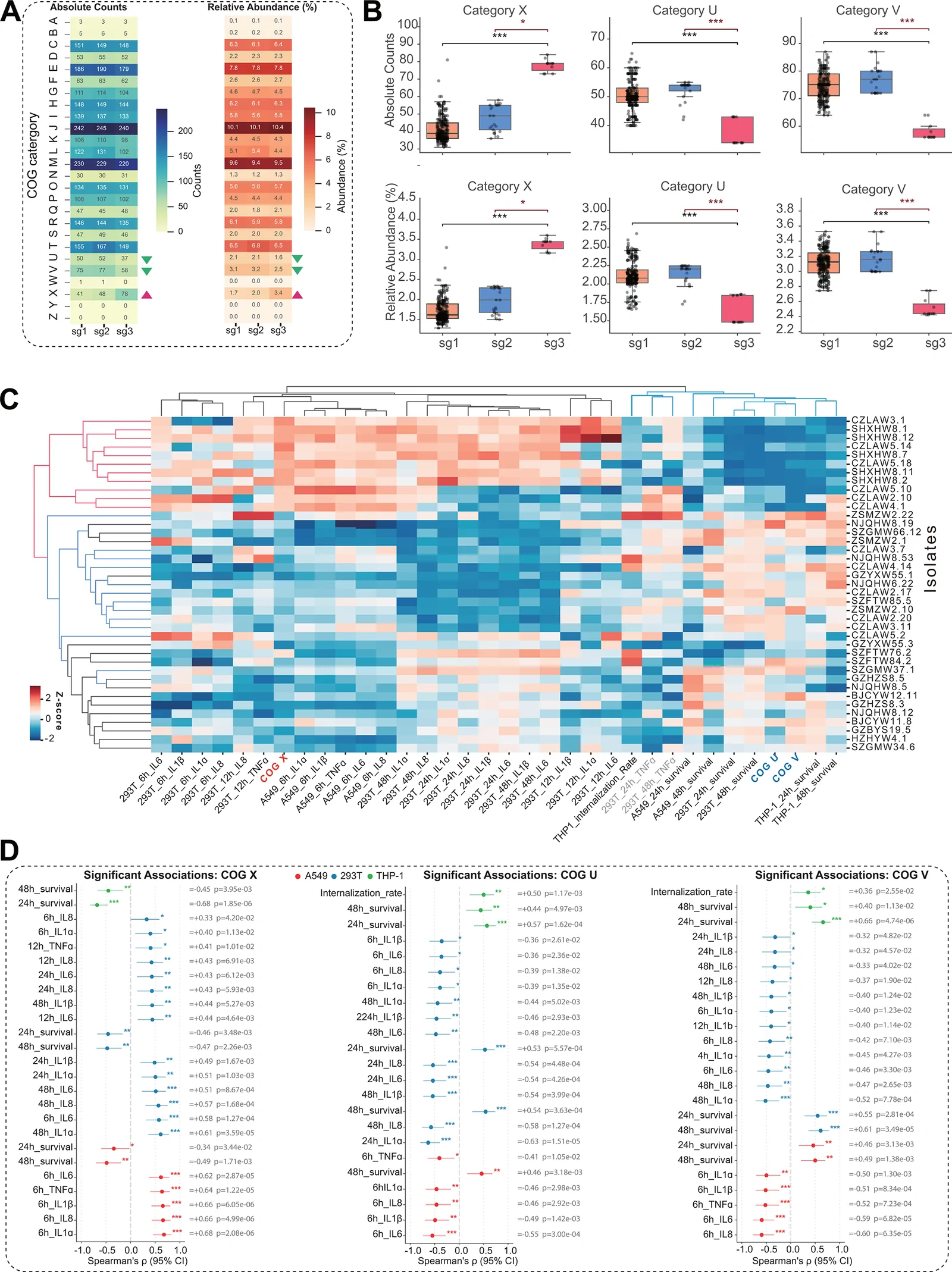
Genomic correlates of serogroup-specific pathogenicity. (**A**) Absolute (left) and relative (right) COG category abundances for sg1, sg2, and putative sg3. Green and purple arrows denote categories significantly reduced or enriched in sg3, respectively. (**B**) Distribution of COG X, COG U, and COG V abundances across serogroups (one-way ANOVA). (**C**) Hierarchical clustering of COG category abundances with phenotypic variables. sg3 isolates cluster separately, with COG X linked to inflammatory readouts and COG U/COG V associated with cell survival. (**D**) Spearman rank correlation matrix of phenotypic and COG variables. For panels B and D: *P < 0.05, **P < 0.01, and ***P < 0.001.

Putative sg3 exhibited a distinct functional signature characterized by a marked expansion of the mobilome (COG X; prophages, transposons, and integrative elements) and a concomitant contraction of genes governing intracellular trafficking, secretion, and vesicular transport (COG U), alongside defense mechanisms (COG V) (Fig. 7A and B). To test whether these broad functional shifts, or classical virulence determinants, underpin the observed phenotypes, we performed two complementary, phylogenetically informed analyses. First, we screened 176 curated virulence factors including known Dot/Icm effectors and surface structures (Table S4), identifying 148 core and 28 lineage-specific loci. Although several virulence factors showed sg3-restricted distributions (Fig. S4), PGLS revealed no significant association between the presence/absence of any individual virulence gene and host-interaction phenotype after FDR correction (all q > 0.99; Table S5). Second, we applied rank-transformed PGLS to COG category abundances. This analysis revealed that COG X abundance was positively associated with two proinflammatory cytokine levels in HEK293T cells after FDR correction: IL1β at 6 h post-infection (coefficient = 4.51, *P* = 0.00017, q = 0.013) and IL1α at 24 h post-infection (coefficient = 2.02, *P* = 0.00028, q = 0.013) (Table S5). A third cytokine, IL6 at 6 h, showed a nominal positive association (coefficient = 2.51, *P* = 0.016) but did not remain significant after FDR correction (q = 0.38). No significant associations were observed for COG U or COG V with any inflammatory endpoint (all q > 0.99; Table S6), a limitation that likely reflects the small number of sg3 isolates (*n* = 11) and their high genetic relatedness, which constrain statistical power. Unsupervised hierarchical clustering of Z-score-normalized variables visually segregated sg3 isolates, with higher COG X abundance clustering with proinflammatory profiles and higher COG U/COG V clustering with host cell survival (Fig. 7C). Spearman correlations also supported these patterns (Fig. 7D).

Taken together, the enhanced inflammatory phenotype of sg3 may not be explained by the gain or loss of individual accessory virulence factors but is instead significantly associated with mobilome (COG X) expansion when monotonic evolutionary relationships are properly accounted for. This suggests that higher-order genomic restructuring, rather than individual effector acquisition, may be associated with the distinct pathogenic strategy of sg3.

## DISCUSSION

Our pan-genomic and functional characterization of 242 *L. longbeachae* isolates reveals substantial genomic plasticity and phenotypic heterogeneity within this emerging pathogen. The identification of a putative novel serogroup (sg3) with distinct genomic architecture, genome reduction, plasmid absence, and enhanced proinflammatory capacity broadens the current understanding of *L. longbeachae* population structure and pathoadaptive strategies.

The open pan-genome (γ = 0.11) places *L. longbeachae* alongside highly fluid bacterial species, comparable to *Streptococcus agalactiae* and *L. pneumophila* (17–19). The large proportion of cloud genes (>40%) and enrichment of mobilome (COG X), replication/recombination (COG L), and defense (COG V) functions in the accessory genome underscore the role of horizontal gene transfer in shaping diversity. This genomic fluidity likely reflects adaptation to complex soil-based environments (compost and potting soils), contrasting with the aquatic niche of *L. pneumophila*. The continued pan-genome expansion suggests that current databases capture only a fraction of *L. longbeachae* diversity, with implications for surveillance.

The discovery of putative sg3 adds a new dimension to our understanding of *L. longbeachae* diversity. Multiple independent lines of evidence support this designation. Previous studies have established a link between *mip* gene polymorphisms and *L. longbeachae* serogroup reactivity (10). The distinct *mip* polymorphisms in the 11 Chinese environmental isolates, together with their unique O-antigen biosynthesis cluster, phylogenomic separation, and phenotypic divergence, support the provisional classification of these isolates as a putative novel serogroup. While the Mip protein is highly conserved between *L. pneumophila* and *L. longbeachae* (~87% amino acid identity, and both proteins consist of 233 amino acids), the sg3-specific *mip* polymorphisms (Fig. 2E) distinguish it from sg1/sg2, highlighting their potential utility as markers for molecular identification of this lineage. Notably, sg3 lacks *orf9* (encoding a putative N-acetyltransferase) in its O-antigen cluster. Since N-acetylation modifications profoundly influence LPS immunogenicity and serological specificity (20–22), this structural variation raises the testable hypothesis that conventional sg1-/sg2-specific assays may exhibit reduced sensitivity for putative sg3. If confirmed, this could have implications for the detection of sg3-associated Legionnaires’ disease, although this remains to be evaluated in clinical diagnostic settings.

Consistent with these findings, exhaustive searches of public genomic databases (e.g., NCBI GenBank) did not identify any additional sg3 lineage isolates, suggesting that this putative serogroup remains underrepresented in current sequence repositories.

Putative sg3 exhibits a reduced genome (4.01 Mb) and complete absence of plasmids, features that define its distinct genomic architecture and evolutionary trajectory. Genome reduction in bacterial pathogens often reflects niche specialization and loss of dispensable functions in stable environments (23–25). Despite its streamlined genome, sg3 does not show attenuated virulence *in vitro*; rather, it displays enhanced cytotoxicity and proinflammatory capacity in specific cell types. This observation indicates that genome reduction in this lineage has not compromised its host interaction capacity; rather, it may coexist with heightened pathogenic phenotypes. However, whether the reduction *per se* contributes to these phenotypes or is simply a correlated feature of lineage divergence cannot be determined from the present data.

Despite the fact that the putative sg3 showed enhanced cytotoxicity in epithelial cells (HEK293T and A549) and macrophages (THP-1), it only induced stronger proinflammatory cytokine responses than sg1/sg2 in HEK293T cells (IL1α, IL1β, IL6, IL8, and TNFα) and early in A549 cells, but not in THP-1 macrophages, revealing cell-type specificity. These findings contrast with the report that *L. longbeachae* is immunologically silent in human pulmonary epithelial cells and macrophages (26), revealing substantial functional divergence among serogroups. The enhanced inflammatory phenotype depended on live bacteria; heat-killed bacteria (70°C, 30 min) induced only low-level, serogroup-independent responses, indicating that active bacterial processes (e.g., Dot/Icm secretion) rather than passive exposure to pathogen-associated molecular patterns (PAMPs) may underlie the observed phenotype (27, 28).

To account for nonlinearity and outliers, we performed rank-transformed PGLS. This revealed a significant positive association between COG X (mobilome) expansion and two proinflammatory cytokines in HEK293T cells after FDR correction: IL1β at 6 h and IL1α at 24 h (q = 0.013). A similar trend for IL6 at 6 h did not survive correction. By contrast, no individual virulence factor showed any significant association. Thus, mobilome enrichment is a consistent genomic correlate of sg3’s enhanced inflammatory phenotype, suggesting that higher-order genomic restructuring, rather than discrete effector acquisition, is associated with its distinct pathogenic strategy.

The cell-type specificity may reflect the differential expression of pattern recognition receptors or inflammasome components. Several speculative mechanisms could be considered to explain sg3’s enhanced inflammation: loss of T4SS effectors (SdjA and Ceg19) may alter vacuole biogenesis (29, 30); the absence of the putatively N-acetyltransferase-encoding orf9 in the O-antigen cluster might influence LPS structure and TLR4/MD-2 activation (31); or sg3-specific effectors (VipF, SodC, and SidG) might directly enhance host responses (32–35). The complete lack of plasmids in sg3 contrasts with their near-ubiquity in sg1/sg2. Plasmid maintenance incurs fitness costs; loss may be favored in stable environments, or essential plasmid-encoded functions may have been chromosomally integrated. Alternatively, plasmid absence could contribute to the inflammatory phenotype by eliminating plasmid-borne immunomodulatory factors.

The existence of putative sg3 highlights the potential need for revised diagnostic algorithms and sg3-inclusive panels, particularly in regions where it has been detected. Its enhanced proinflammatory capacity raises questions about its clinical significance, warranting further clinical studies. Several limitations merit note: *in vitro* models cannot fully recapitulate *in vivo* complexity; bacterial transcriptomics would provide mechanistic insights; the limited number (*n* = 11) and high genetic similarity of sg3 isolates likely constrain genotype-phenotype associations. Expanding sg3 diversity and performing targeted gene knockouts are essential next steps.

Future research directions should prioritize structural characterization of sg3 LPS, systematic screening of sg3-specific effectors, studies using primary human cells and organoids and animal models, development of sg3-specific diagnostic reagents, and clinical studies to define its epidemiological significance.

### Conclusions

Our study reveals unexpected genomic and phenotypic diversity within *L. longbeachae*, identifying a putative novel serogroup that combines enhanced cytotoxicity to human cell lines and proinflammatory capacity in epithelial-derived cells with a distinctive genomic architecture marked by genome reduction and mobilome expansion. These findings call into question the conventional views of genome size-virulence relationships in the context of *in vitro* infection models and underscore the need for continued genomic surveillance.

## MATERIALS AND METHODS

### Genome data acquisition and sequencing

Eleven reference-quality genome assemblies were retrieved from the NCBI GenBank database, and 388 raw read data sets were downloaded from the NCBI Sequence Read Archive (SRA), with data acquisition concluded in December 2025. To complement these public resources and capture regional diversity, we performed *de novo* whole-genome sequencing of 39 environmental *L. longbeachae* isolates. These isolates were recovered from environmental samples collected during our routine environmental surveillance programs across diverse niches in China, including soil, potting mixes, and water sources from multiple provinces (36). Long-read sequencing was conducted using Oxford Nanopore Technologies (ONT) on the GridION platform (IGE Biotechnology, Guangzhou, China). For SRA-derived data sets, raw reads underwent stringent quality control and adapter trimming using fastp (37), followed by *de novo* assembly with Unicycler (38).

### Genome quality control and data set curation

All genomes were assessed with checkM v1.2.3 (39) and checkM2 (40). To ensure the reliability of downstream comparative and functional analyses, stringent quality control thresholds were applied. Assemblies were excluded if they failed to meet any of the following criteria: (i) genome completeness <90% (CheckM) or <95% (CheckM2); (ii) contamination >10% (CheckM) or >5% (CheckM2); or (iii) contig/scaffold N50 <100 kbp. The final curated set comprised 242 high-quality, nonredundant genomes (Table S1).

### *L. longbeachae* pangenome construction

All assemblies were annotated with Bakta v1.11.4 (41) using standardized bacterial parameters. This pipeline generated structured GFF3 annotations encompassing protein-coding sequences, noncoding RNAs, and database-derived functional assignments. Pangenome inference was performed with Panaroo v1.5.1 (42) using ≥98% identity and ≥95% alignment coverage. Gene clusters were classified as core (≥99% of isolates), soft-core (95%–99%), shell (≥15% to <95%), or cloud (<15%). The primary output comprised a binary presence-absence matrix (isolates × gene clusters), which served as the foundational feature space for downstream comparative and statistical analyses. To characterize pangenome dynamics, accumulation curves for both the pangenome and core genome were generated using a custom Python-based rarefaction framework. Briefly, the genomic cohort was randomly subsampled without replacement across 242 independent permutations. At each subsampling step, the mean number of total and shared gene clusters was computed. The pangenome size trajectory was subsequently fitted to Heaps’ law [𝑇(𝑛)=*k*𝑛𝛾], where the exponent 𝛾 quantitatively defines the openness of the pangenome.

### Genomic relatedness and population structure analysis of *L. longbeachae* isolates

We employed the preprocessing module of the Pan-Genome Analysis Pipeline 2 (PGAP2) to perform standardized comparative annotation (40). Using the representative strain NCTC11477 (GenBank accession: GCA_004283175.1) as a reference, we calculated average nucleotide identity (ANI) values for each isolate to quantify sequence-level divergence. Concurrently, we extracted gene content metrics for each genome, including total protein-coding gene counts and the number of “half-core” genes, defined as orthologous clusters present in ≥50% of the 242 isolates. We plotted the abundance of half-core pre-clusters against “single-cloud” pre-clusters (orthologous groups unique to a single strain). Isolates exhibiting outlier positions in this bivariate space, particularly those displaced toward the upper-right quadrant, were interpreted as genomically divergent lineages warranting further phylogenetic scrutiny. To resolve fine-scale genetic relationships independent of reference bias, we computed all-versus-all ANI values across the 242-genome data set using fastANI v1.34 (43). Hierarchical clustering (Ward’s method) was applied to the symmetric ANI matrix. *In silico* DNA-DNA hybridization (iDDH) was computed using the GGDC (44, 45).

### Plasmid detection and functional annotation

Plasmid status (presence, absence, and size) was determined using geNomad (version 1.8.0) with default parameters (46). For virulence factor profiling, assembled genomes were aligned against the Virulence Factor Database (VFDB) using ABRicate (47, 48). A minimum sequence coverage threshold of 30% and a minimum nucleotide identity threshold of 60% were applied to balance sensitivity and specificity, as previously recommended (49–52). Functional annotation of orthologous clusters was performed using COGclassifier (https://github.com/moshi4/COGclassifier).

### *L. longbeachae* serogroup identification, molecular typing, and phylogenetic analyses

Serogroup assignment integrated the following: (i) public strain metadata, (ii) *mip* allelic profiling (10), and (iii) O-antigen cluster structure (comparative synteny and functional annotation). Phylogenetic relationships were inferred using a maximum-likelihood approach implemented in IQ-TREE v3.0.1 (53). Core-genome phylogeny was inferred from 2,820 single-copy orthologs (80,888 informative SNPs), with ModelFinder and 1,000 ultrafast bootstraps. Pan-genome phylogeny was inferred from the binary gene presence/absence matrix using a binary substitution model. Both phylogenetic trees were visualized and annotated using the tvBOT web server (54). Collinearity of the conserved and highly orthologous genomic regions was determined and plotted among sg1, sg2, and putative sg3 isolates by using Mauve software (version 2.3.1) with default parameters (55).

### Comparative *in vitro* phenotyping of *L. longbeachae* serogroups in human cell models

A panel of 39 environmental isolates recovered from geographically and ecologically disparate niches across China was stratified into three phylogenetically and antigenically distinct lineages: serogroup 1 (sg1; *n* = 16), serogroup 2 (sg2; *n* = 12), and putative sg3 (*n* = 11). All isolates were propagated on buffered charcoal yeast extract (BCYE) agar at 37^o^C for 72 h to achieve the post-exponential growth phase, a standard growth stage for *Legionella* infection assays in which the Dot/Icm type IV secretion system is known to be highly expressed. Bacterial suspensions were harvested in sterile endotoxin-free water, and inoculum densities were standardized spectrophotometrically at an optical density of 600 nm (OD_600_) to the target multiplicity of infection (MOI). For heat-killing controls, bacterial suspensions were heated at 70°C for 30 min, and complete loss of viability was confirmed by plating 100 μL of the heat-treated suspension onto BCYE agar and incubating at 37°C for up to 7 days, with daily monitoring. Heat-killed bacteria were used at the same multiplicity of index (MOI) (10) as live bacteria in the cytotoxicity and cytokine assays.

To recapitulate the cellular tropism and tissue-contextualized host-pathogen interfaces relevant to *L. longbeachae* infection, three human cell lines representing distinct infection niches were employed. THP-1 monocytes (ATCC TIB-202), differentiated into macrophage-like cells, served as a model of the alveolar macrophage, the principal intracellular replicative niche and primary target cell of *Legionella* species in the human lung. A549 cells (ATCC CCL-185), derived from type II alveolar epithelium, were utilized as a physiologically relevant model of the pulmonary epithelial barrier, which constitutes a secondary, clinically implicated target of *Legionella* infection. HEK293T cells (ATCC CRL-3216), a well-characterized embryonic kidney epithelial line, were included as a generic epithelial cell platform to discriminate lung-epithelium-restricted responses from broader, cell type-independent epithelial interaction phenotypes. THP-1 cells were maintained in RPMI 1640 medium, while A549 and HEK293T cells were cultured in Dulbecco’s modified Eagle’s medium (DMEM). All basal media were supplemented with 10% (vol/vol) heat-inactivated fetal bovine serum, 2 mM L-glutamine, and 1% (vol/vol) penicillin-streptomycin. Cultures were maintained at 37°C in a humidified atmosphere of 5% CO_2_. Prior to infection, the culture medium was replaced with antibiotic-free medium to prevent interference with bacterial viability during the infection assays. The same antibiotic-free medium was used throughout all infection experiments (internalization, intracellular replication, cytotoxicity, and cytokine assays).

Prior to infection, THP-1 monocytes were differentiated into adherent macrophage-like cells by treatment with 50 ng mL⁻¹ phorbol 12-myristate 13-acetate (PMA) (Biosharp, cat no. BL1127A) for 48 h. Following differentiation, cells were washed three times with phosphate-buffered saline (PBS) and rested for a further 24 h in PMA-free complete medium to allow stabilization of the basal inflammatory state and to mitigate residual PMA-induced activation. Cell line identity was authenticated by short tandem repeat (STR) profiling, and culture sterility was confirmed by routine mycoplasma screening throughout the experimental period.

For infection assays, host cells were seeded into 24-well tissue culture plates at densities of 5 × 10^5^ cells per well for THP-1 macrophages and 1 × 10^5^ cells per well for A549 and HEK293T epithelial cells and subsequently challenged with *L. longbeachae* isolates at an MOI of 10. Bacterial internalization was quantified at 2 h post-infection (hpi) using a flow cytometry-based fluorescence quenching assay. In brief, bacteria were covalently labeled with 500 µg mL⁻¹ fluorescein isothiocyanate (FITC) (Sigma-Aldrich, cat no. F7250) in 0.1 M NaHCO_3_ buffer (pH 9.0) for 30 min at 4°C in the dark, and unbound fluorochrome was removed by extensive washing. Following a 2-h coincubation period, extracellular and surface-adherent bacteria were quenched by exposure to 0.25% trypan blue (Beyotime, cat no. ST798) for 2 min. Cells were then harvested, and the frequency of FITC^+^ events—representing internalized bacteria—was acquired on a CytoFLEX LX flow cytometer. Internalization efficiency was normalized to the initial bacterial inoculum corresponding to the applied MOI of 10, thereby accounting for potential dose-dependent variation in uptake.

Intracellular replication kinetics were profiled over a 48-h time course. At 6, 12, 24, and 48 hpi, infected monolayers were washed three times with PBS to remove non-internalized bacteria and lysed with sterile endotoxin-free water. Lysates were serially diluted, plated onto BCYE agar, and colony-forming units (CFU) were enumerated following incubation at 37°C for 3–5 days. Replication fold-change was calculated by normalizing CFU counts at each time point to the 6-h baseline, a reference time point empirically defined as corresponding to the completion of phagosomal escape and establishment of productive intracellular replication.

Infection-induced cytotoxicity was assessed longitudinally by dual-parameter flow cytometry using an annexin V–FITC / propidium iodide (PI) apoptosis detection kit (Biosharp, cat no. BL107B), performed according to the manufacturer’s protocol. At 6, 12, 24, and 48 hpi, both adherent and detached cells were collected, washed in cold PBS, and resuspended in annexin-binding buffer. Following dual-fluorescence staining, cell populations were resolved into four quadrants representing distinct viability states: viable (annexin V⁻ PI⁻), early apoptotic (annexin V^+^ PI⁻), late apoptotic/secondary necrotic (annexin V^+^ PI^+^), and necrotic (annexin V⁻ PI^+^). The percentage of viable cells was used as the primary endpoint for comparative cytotoxicity profiling across serogroups.

Host inflammatory responses were characterized by quantifying the transcriptional kinetics of a curated panel of proinflammatory cytokines, IL1α, IL1β, IL6, IL8,, IL10 and TNFα via quantitative reverse transcription PCR (qRT-PCR). Total RNA was extracted at 6, 12, 24, and 48 hpi (Vazyme, cat no. RC112-01) and reverse-transcribed into cDNA using a high-capacity cDNA synthesis kit (Vazyme, cat no. R333-C1). Quantitative PCR was performed with SYBR Green master mix on a CFX96 Touch real-time PCR detection system (Biosharp, cat no. BL697A). Thermal cycling conditions comprised an initial denaturation at 95 °C for 2 min, followed by 40 cycles of 95°C for 15 s and 60°C for 20 s. Primer sequences are provided in Table 1. Relative gene expression was normalized to the constitutively expressed reference gene GAPDH and calculated using the comparative 2⁻ΔΔCt method.

**TABLE 1:** qPCR primer sequences used in this study

Primer	Sequence (5′ to 3′)	Primer target (accession no.)	Product length (bp)
IL1ɑ-F	TGTATGTGACTGCCCAAGATGAAG	NM_000575	96
IL1ɑ-R	AGAGGAGGTTGGTCTCACTACC		
IL1β-F	CCACAGACCTTCCAGGAGAATG	NM_000576	131
IL1β-R	GTGCAGTTCAGTGATCGTACAGG		
IL6_F	AGACAGCCACTCACCTCTTCAG	NM_000600	132
IL6_R	TTCTGCCAGTGCCTCTTTGCTG		
IL8_F	GAGAGTGATTGAGAGTGGACCAC	NM_000584	112
IL8_R	CACAACCCTCTGCACCCAGTTT		
IL10_F	TCTCCGAGATGCCTTCAGCAGA	NM_000572	126
IL10_R	TCAGACAAGGCTTGGCAACCCA		
TNFɑ_F	CTCTTCTGCCTGCTGCACTTTG	NM_000594	135
TNFɑ_R	ATGGGCTACAGGCTTGTCACTC		
GAPDH_F	GTCTCCTCTGACTTCAACAGCG	NM_002046	131
GAPDH_R	ACCACCCTGTTGCTGTAGCCAA		

For each of the 39 isolates, infection assays (internalization, intracellular replication, cytotoxicity, and cytokine profiling) were performed in three culture wells, using the same bacterial inoculum and cell preparation process. The three replicate measurements were averaged to obtain a single value per isolate. These isolate-level means were then used as independent observations for serogroup-level statistical comparisons (sg1, *n* = 16; sg2, *n* = 12; putative sg3, *n* = 11).

### Statistical analysis

Data are presented as mean ± standard error of the mean (SEM) of isolate-level means. Serogroup-dependent differences in each phenotypic variable (bacterial internalization, intracellular replication, cytotoxicity, and individual cytokine levels) were assessed by one-way analysis of variance (ANOVA), followed by Tukey’s *post hoc* test. For pairwise exploratory associations analysis, Spearman’s rank correlation coefficients (ρ) were calculated between COG category abundances and phenotypic variables (bacterial internalization, cytotoxicity, and cytokine levels) without phylogenetic correction, using individual isolates as independent data points. These non-phylogenetic correlations were used for exploratory visualization and hypothesis generation. To account for phylogenetic relatedness, we performed rank-transformed PGLS regression, which is equivalent to a phylogenetically corrected Spearman rank correlation. Both COG abundances and phenotypic variables were rank-transformed prior to analysis. The phylogenetic covariance matrix was constructed under a Brownian motion model (λ = 1), and the tree was midpoint-rooted. *P*-values were adjusted for multiple testing using the Benjamini-Hochberg false discovery rate (FDR) method. All statistical computations were carried out in GraphPad Prism (v8.0.2) and Python 3.12 (with SciPy 1.16.0 and statsmodels 0.14.5). Statistical significance was defined as *P* < 0.05.

## Data Availability

The complete whole-genome sequences of the 39 representative Chinese environmental *L. longbeachae* isolates have been deposited in the National Center for Biotechnology Information (NCBI) database and are available under BioProject accession number PRJNA1267263. All public assemblies used are listed with accessions in Table S1, and all phenotypic and statistical data supporting the conclusions are provided in the article and supplemental tables.

## SUPPLEMENTAL MATERIAL

Supplemental figures aem.01109-26-s0001.pdf Fig. S1 to S4.

Table S1 aem.01109-26-s0002.xlsx *L. longbeachae* isolate information.

Table S2 aem.01109-26-s0003.docx iDDH values of putative sg3 isolates against the *L. longbeachae* type strain NCTC11477.

Table S3 aem.01109-26-s0004.xlsx Plasmid sequences detected in *L. longbeachae* isolates across different serogroups.

Table S4 aem.01109-26-s0005.xlsx Curated list of virulence factors used in this study, including Dot/Icm effectors and surface structures, with presence/absence across *L. longbeachae* isolates.

Table S5 aem.01109-26-s0006.xlsx PGLS regression results for associations between individual virulence genes and host‑interaction phenotypes across *L. longbeachae* isolates.

Table S6 aem.01109-26-s0007.xlsx PGLS regression results for associations between COG category abundances (COG U, COG V, and COG X) and host‑interaction phenotypes across *L. longbeachae* isolates.

ASM does not own the copyrights to Supplemental Material that may be linked to, or accessed through, an article. The authors have granted ASM a non-exclusive, world-wide license to publish the Supplemental Material files. Please contact the corresponding author directly for reuse.
