# Supplementary material for: Genomic divergence and phenotypic heterogeneity of Legionella longbeachae reveal a putative novel proinflammatory serogroup with genome reduction and mobilome expansion
Source: Appl Environ Microbiol. 2026 Sep 22;92(10):e01109-26. doi: 10.1128/aem.01109-26 (PMC13689119; doi:10.1128/aem.01109-26)
Supplement: Supplementary file 1 [file aem.01109-26-s0001.pdf]

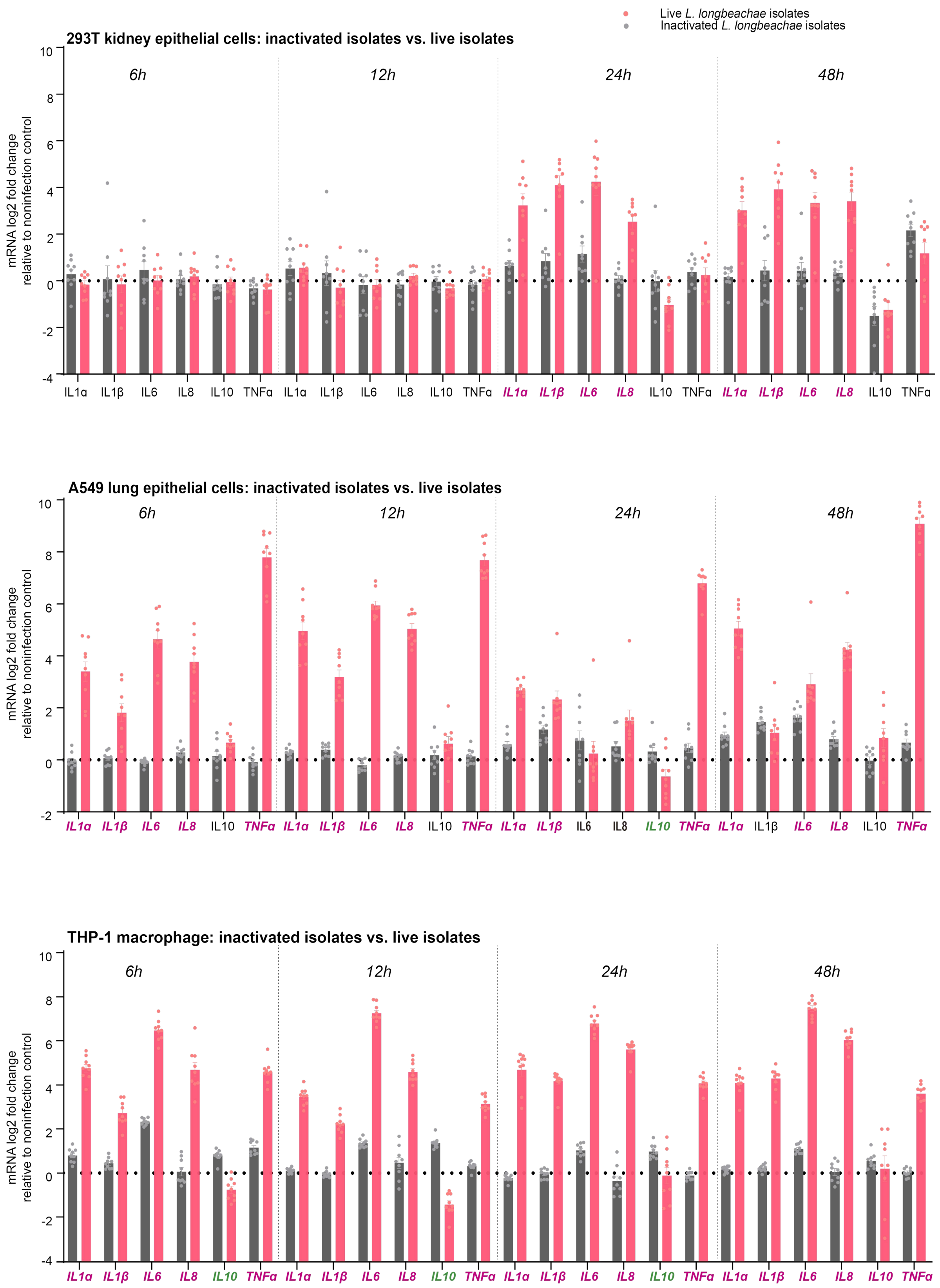

Live L. longbeachae isolates
Inactivated L. longbeachae isolates
293T kidney epithelial cells: inactivated isolates vs. live isolates
6h
12h
24h
48h
mRNA log2 fold change relative to noninfection control
IL1α IL1β IL6 IL8 IL10 TNFα IL1α IL1β IL6 IL8 IL10 TNFα IL1α IL1β IL6 IL8 IL10 TNFα IL1α IL1β IL6 IL8 IL10 TNFα
A549 lung epithelial cells: inactivated isolates vs. live isolates
6h
12h
24h
48h
mRNA log2 fold change relative to noninfection control
IL1α IL1β IL6 IL8 IL10 TNFα IL1α IL1β IL6 IL8 IL10 TNFα IL1α IL1β IL6 IL8 IL10 TNFα IL1α IL1β IL6 IL8 IL10 TNFα
THP-1 macrophage: inactivated isolates vs. live isolates
6h
12h
24h
48h
mRNA log2 fold change relative to noninfection control
IL1α IL1β IL6 IL8 IL10 TNFα IL1α IL1β IL6 IL8 IL10 TNFα IL1α IL1β IL6 IL8 IL10 TNFα IL1α IL1β IL6 IL8 IL10 TNFα

Figure S1 **Inflammatory responses depend on live bacterial infection.** mRNA expression levels of inflammation-related factors in 293T, A549 and THP-1 cells infected with live (red dots) or heat-inactivated (grey dots) *L. longbeachae* isolates (nine isolates total, three per serogroup) at 6, 12, 24 and 48 h. Heat-inactivated bacteria generally failed to induce significant cytokine upregulation relative to uninfected controls (black dashed line, $\log_2$ fold change = 0). Live infection actively suppressed *IL10* expression compared with heat-inactivated bacteria at specific time points. Cytokines shown in magenta italics were significantly upregulated in the live group versus the inactivated group, whereas those in green italics were significantly downregulated (paired t-test).

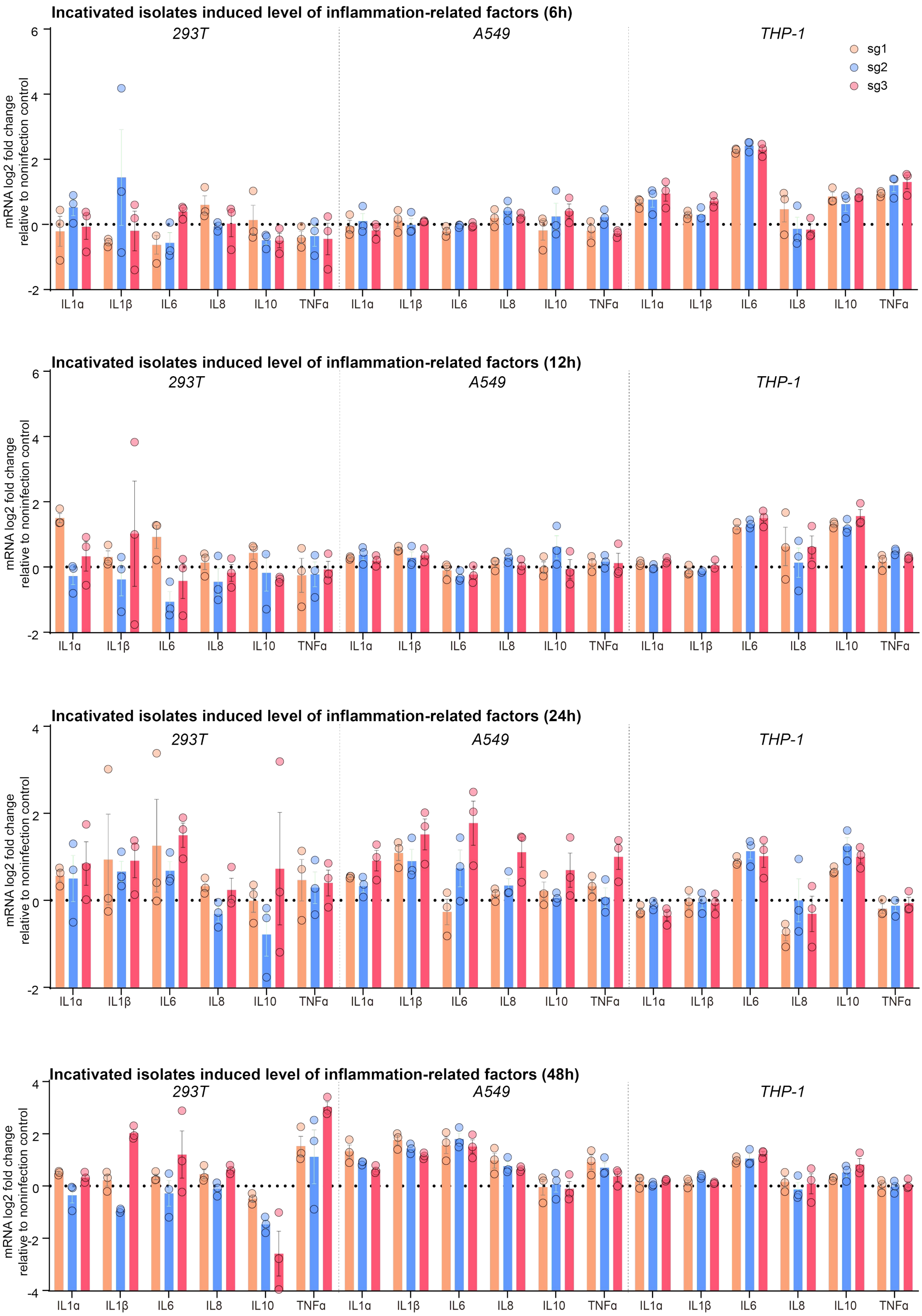
Incativated isolates induced level of inflammation-related factors (6h)
293T
A549
THP-1
sg1
sg2
sg3
mRNA log2 fold change relative to noninfection control
6
4
2
0
-2
IL1α IL1β IL6 IL8 IL10 TNFα IL1α IL1β IL6 IL8 IL10 TNFα IL1α IL1β IL6 IL8 IL10 TNFα
Incativated isolates induced level of inflammation-related factors (12h)
293T
A549
THP-1
mRNA log2 fold change relative to noninfection control
6
4
2
0
-2
IL1α IL1β IL6 IL8 IL10 TNFα IL1α IL1β IL6 IL8 IL10 TNFα IL1α IL1β IL6 IL8 IL10 TNFα
Incativated isolates induced level of inflammation-related factors (24h)
293T
A549
THP-1
mRNA log2 fold change relative to noninfection control
4
2
0
-2
IL1α IL1β IL6 IL8 IL10 TNFα IL1α IL1β IL6 IL8 IL10 TNFα IL1α IL1β IL6 IL8 IL10 TNFα
Incativated isolates induced level of inflammation-related factors (48h)
293T
A549
THP-1
mRNA log2 fold change relative to noninfection control
4
2
0
-2
-4
IL1α IL1β IL6 IL8 IL10 TNFα IL1α IL1β IL6 IL8 IL10 TNFα IL1α IL1β IL6 IL8 IL10 TNFα

Figure S2 **Heat-inactivated isolates of different serogroups induce comparably negligible inflammatory responses.** Comparison of mRNA expression levels of inflammation-related factors in cells treated with heat-inactivated representative isolates of sg1 (brown), sg2 (blue), and putative sg3 (red) at 6, 12, 24, and 48 h post-treatment. Three representative isolates per serogroup were used in this control experiment. No significant differences in cytokine induction were observed among the serogroups (two-sided t-tests).

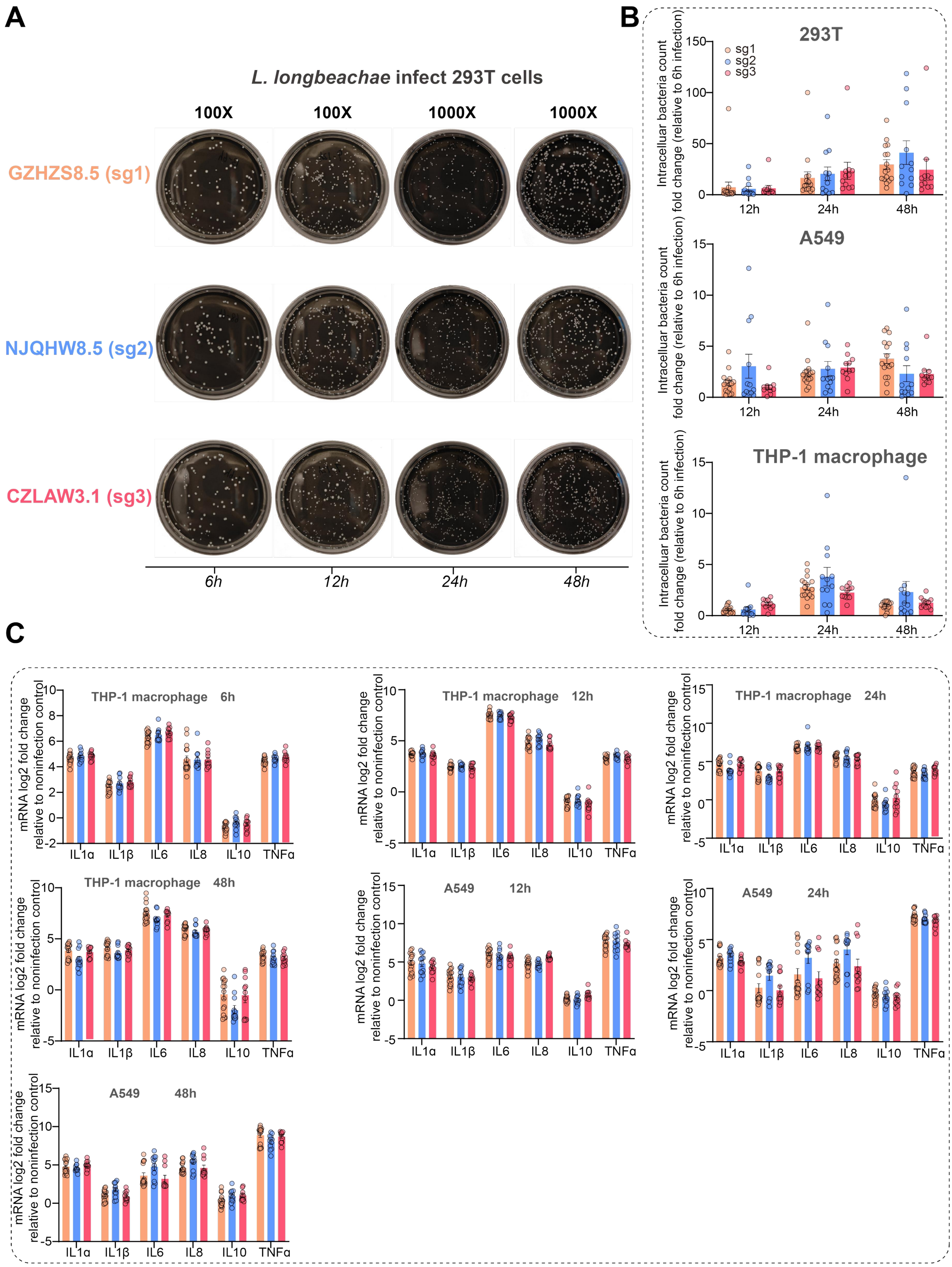
A
L. longbeachae infect 293T cells
100X
100X
1000X
1000X
GZHZS8.5 (sg1)
NJQHW8.5 (sg2)
CZLAW3.1 (sg3)
6h
12h
24h
48h
B
293T
A549
THP-1 macrophage
sg1
sg2
sg3
Intracelluar bacteria count fold change (relative to 6h infection)
12h
24h
48h
C
THP-1 macrophage 6h
THP-1 macrophage 12h
THP-1 macrophage 24h
THP-1 macrophage 48h
A549 12h
A549 24h
A549 48h
mRNA log2 fold change relative to noninfection control
IL1α
IL1β
IL6
IL8
IL10
TNFα

Figure S3 **Intracellular proliferation kinetics and host inflammatory responses across *L. longbeachae* serogroups.** **A**, Representative CFU plates from lysates of 293T cells infected with representative isolates of serogroup 1 (GZHZS8.5), serogroup 2 (NJQHW8.5), and serogroup 3 (CZLAW3.1). Cells were lysed at indicated time points (6–48 h) and plated at 100× and 1000× dilutions. **B**, Quantification of intracellular bacterial proliferation. Fold change in intracellular bacterial counts relative to 6 h post-infection in 293T, A549, and THP-1 cells. Statistical analysis (One-way ANOVA) revealed no significant differences in replication rates among serogroups across all cell lines. Data are presented as mean ± SEM. **C**, mRNA expression levels of inflammatory cytokines (IL1α, IL1β, IL6, IL8, IL10, TNFα) relative to non-infection controls. Top panels: THP-1 macrophages at 6, 12, 24, and 48 h post-infection. Bottom panels: A549 cells at 12, 24, and 48 h post-infection. No significant differences in cytokine induction were observed between serogroups in THP-1 cells at any time point, or in A549 cells at later time points (12–48 h). Sample sizes: Sg1 (n = 16), Sg2 (n = 12), putative Sg3 (n = 11).

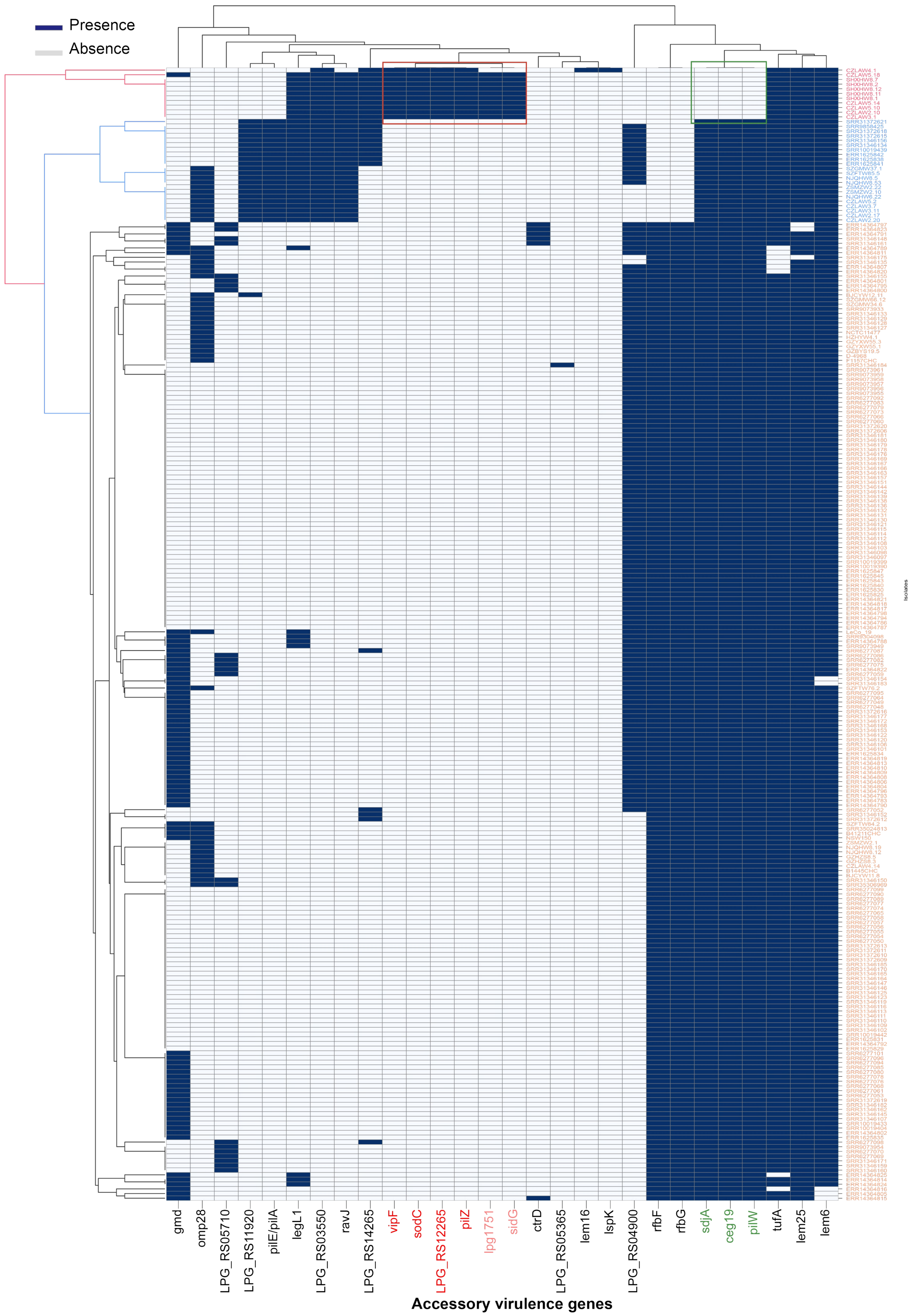
Presence
Absence
gmd
omp28
LPG_RS05710
LPG_RS11920
pilE/pilA
legL1
LPG_RS03550
ravJ
LPG_RS14265
vipF
sodC
LPG_RS12265
pilZ
lpg1751
sidG
ctrD
LPG_RS05365
lem16
lspK
LPG_RS04900
rfbF
rfbG
sdjA
ceg19
pilW
tufA
lem25
lem6
Accessory virulence genes

Figure S4 **Comparative analysis of accessory virulence factor distribution.** The heatmap depicts the presence (dark blue) and absence (light grey) of accessory virulence genes across *L. longbeachae* isolates. Genes were defined as accessory if they were not present in 100% of the population. Hierarchical clustering based on binary distance metrics was applied to both isolates (rows) and genes (columns), revealing a distinct segregation of the accessory genome profiles. Isolates are color-coded by serogroup: sg1 (brown), sg2 (blue), and putative sg3 (red). The analysis demonstrates that sg3 forms a distinct clade characterized by a unique virulence repertoire. Specifically, sg3 isolates harbor a distinct subset of T4SS effectors and pilus biogenesis genes (labelled in red, e.g., *vipF*, *sodC*, *LPG_RS12265*, *pilZ*, *lpg1751*, *sidG*) while lacking specific T4SS effectors and pilus proteins (labelled in green, e.g., *sdjA*, *ceg19*, *pilW*) that are conserved in sg1 and sg2 lineages.
