# Supplementary material for: Genomic divergence and phenotypic heterogeneity of Legionella longbeachae reveal a putative novel proinflammatory serogroup with genome reduction and mobilome expansion
Source: Appl Environ Microbiol. 2026 Sep 22;92(10):e01109-26. doi: 10.1128/aem.01109-26 (PMC13689119; doi:10.1128/aem.01109-26)
Supplement: Supplementary file 2 [file aem.01109-26-s0003.docx]

**Table S2. iDDH values of putative sg3 isolates against the *L. longbeachae* type strain NCTC11477.**

| **Query strain** | **Subject strain** | **iDDH (d0, in %)** | **95% C.I. (d0, in %)** | **iDDH (d4, in %)*** | **95% C.I. (d4, in %)** | **iDDH (d6, in %)** | **95% C.I. (d6, in %)** | **G+C content difference (in %)** |
| --- | --- | --- | --- | --- | --- | --- | --- | --- |
| NCTC11477 | CZLAW2.10 | 86.1 | [82.5 - 89.1%] | 70.6 | [67.6 - 73.4%] | 86.2 | [83.1 - 88.8%] | 0.17 |
| NCTC11477 | CZLAW3.1 | 85.6 | [81.9 - 88.6%] | 70.8 | [67.8 - 73.6%] | 85.8 | [82.7 - 88.5%] | 0.19 |
| NCTC11477 | CZLAW4.1 | 86.1 | [82.4 - 89.1%] | 70.9 | [67.9 - 73.8%] | 86.3 | [83.1 - 88.9%] | 0.16 |
| NCTC11477 | CZLAW5.10 | 86.1 | [82.5 - 89.1%] | 70.6 | [67.6 - 73.4%] | 86.2 | [83.1 - 88.8%] | 0.17 |
| NCTC11477 | CZLAW5.14 | 85.6 | [81.9 - 88.6%] | 70.8 | [67.8 - 73.7%] | 85.8 | [82.7 - 88.5%] | 0.18 |
| NCTC11477 | CZLAW5.18 | 86.5 | [82.8 - 89.4%] | 70.8 | [67.8 - 73.6%] | 86.5 | [83.5 - 89.1%] | 0.16 |
| NCTC11477 | SHXHW8.1 | 86.3 | [82.7 - 89.3%] | 70.8 | [67.8 - 73.7%] | 86.4 | [83.4 - 89%] | 0.16 |
| NCTC11477 | SHXHW8.2 | 86.3 | [82.7 - 89.3%] | 70.8 | [67.8 - 73.7%] | 86.4 | [83.4 - 89%] | 0.16 |
| NCTC11477 | SHXHW8.7 | 86.3 | [82.7 - 89.3%] | 70.8 | [67.8 - 73.7%] | 86.4 | [83.4 - 89%] | 0.16 |
| NCTC11477 | SHXHW8.11 | 86.3 | [82.7 - 89.3%] | 70.8 | [67.8 - 73.7%] | 86.4 | [83.4 - 89%] | 0.16 |
| NCTC11477 | SHXHW8.12 | 86.3 | [82.7 - 89.3%] | 70.8 | [67.8 - 73.7%] | 86.4 | [83.4 - 89%] | 0.16 |

*Formula *d_4_* (i.e., GGDC formula 2) is defined as the sum of all identities in high-scoring segment pairs (HSPs) divided by the total length. It yields more robust results and is therefore recommended as the standard.
